# Méningo-vascularite bactérienne révélant un adénome hypophysaire

**DOI:** 10.11604/pamj.2015.20.7.5271

**Published:** 2015-01-05

**Authors:** Siham Bouchal, Hanane Razzouki, Salma Ibn Elkhyat, Mustafa Maaroufi, Ouarda El Ouali, Ouafae Messouak, Mohammed Faouzi Belahsen

**Affiliations:** 1 Service de Neurologie, CHU Hassan II Fès Maroc; 2 Service de Radiologie, CHU Hassan II Fès Maroc

**Keywords:** Adénome hypophsaire, brèche ostéoméningée, méningo-vacularite, pituitary adenoma, osteo-meningeal breach, meningo-vasculitis

## Abstract

Un macroadénome à prolactine se présente généralement par un syndrome hormonal associé à un syndrome tumoral. Une méningite bactérienne compliquant un macroprolactinome en dehors de toute thérapie médicale ou chirurgicale est rarement rapporté dans la littérature. Mme R.C âgée de 48 ans s'est présentée aux urgences pour trouble de conscience fébrile. La ponction lombaire a révélé une méningite bactérienne. L'imagerie cérébrale a mis en évidence un aspect de vascularite cérébrale et un processus de la loge sellaire avec lyse du plancher faisant évoquer une brèche ostéoméningée. Le bilan biologique a montré une hyperprolactinémie à 200 mg/dl. La patiente est mise sous antibiothérapie à dose méningée et une corticothérapie associée à un traitement par la Cabergoline. Le traitement chirurgical de la brèche ostéoméningée s'est fait par voie endonasale. L’évolution est marquée par une nette amélioration clinique et biologique et l'absence de récidive de la méningite après un recul de 14 mois. Un macroprolactinome peut provoquer une brèche ostéoméningée en dehors de tout traitement médical ou chirurgical et avoir comme première manifestation une méningite infectieuse.

## Introduction

Les manifestations classiques révélant un adénome hypophysaire sont représentées par l'association d'un syndrome tumoral (céphalées, troubles visuels…) et d'un syndrome endocrinien. Dans un contexte d'adénome hypophysaire, la méningite bactérienne est une complication possible du traitement chirurgical ou médical. Elle est expliquée par l'existence d'une brèche ostéoméningée provoquée par l'adénome. Une méningite bactérienne est une complication possible après la chirurgie de l'adénome. La survenue d'une méningite avant tout traitement est exceptionnelle. Nous présentons l'observation d'une méningite compliquée d'une vascularité cérébrale révélant un macroadénome à prolactine jusque là méconnu [1, 2].

## Patient et observation

**Figure 1:**
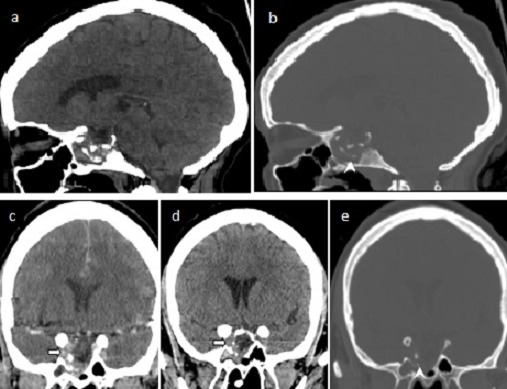
TDM cérébrale avant et après injection de produit de contraste en reconstructions sagittales (a-b) et coronales (c-d-e) montrant un processus tissulaire intrasellaire latéralisé à droite avec extension caverneuse (flèche) et lyse du plancher sellaire avec extension dans le sinus sphénoïdal (tête de flèche)

Mme R.C âgée de 48 ans, admise aux urgences pour agitation et des troubles de comportement dans un contexte fébrile. Elle a comme antécédent une notion d’écoulement nasal clair, épisodique depuis 03 ans associé à des céphalées chroniques modérées. Il n'existait pas de notion de galactorrhée ni d'autres signes d'insuffisance antéhypophysaire. On retrouvait par contre une stérilité primaire. L'examen à son admission trouvait une patiente agitée, confuse, un GCS à 13, fébrile à 39°C, un syndrome méningé franc, et une mydriase aréactive gauche. Elle mobilisait les 4 membres de façon symétrique. Une TDM cérébrale avant et après injection de produit de contraste révèle un processus sellaire avec lyse de la base de crâne (Figure 1).

**Figure 2:**
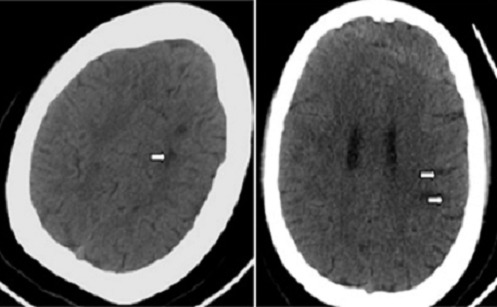
TDM cérébrale montrant des lésions hypodenses multiples sous corticales hémisphériques gauches

**Figure 3:**
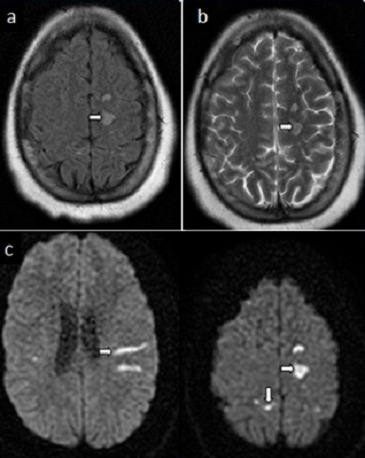
IRM cérébrale en séquence T2(a), FLAIR(b), et diffusion(c) a révélé plusieurs lésions (flèches) en corticales et sous-corticales temporo-fronto-pariétales gauches et temporo-pariétales droites, hyperintenses, punctiformes et linéaires de nature ischémique et de systématisation artérielle

**Figure 4:**
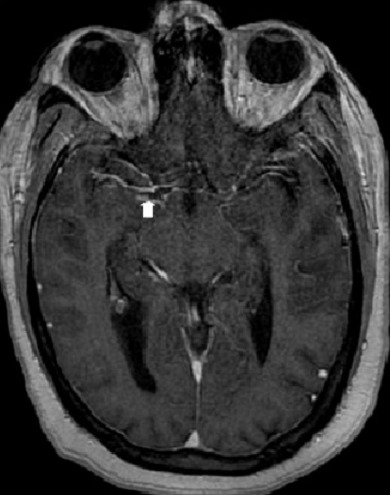
IRM cérébrale montre une prise de contraste de la paroi artérielle de l'artère cérébrale moyenne (Flèche) de façon bilatérale en faveur d'une vascularite

La ponction lombaire ramène un liquide céphalorachidien (LCR) trouble avec à son étude cytologique une méningite à 8000 globules blancs/mm^3^ à prédominance polynucléaires neutrophiles (90%). L'examen direct retrouve un cocci gram positif. L'examen chimique retrouve une hyperprotéinorachie à 5.94 g/l et une hypoglycorrachie à 0.01g/l. La culture bactérienne objective un Streptococcus mitis. La protéine C réactive (CRP) était élevée à 300mg/L et une hyperleucocytose dans le sang à 27.690 éléments/mm^3^. Devant ce tableau de méningite purulente compliquée de trouble de conscience, la patiente est traitée par une céphalosporine de 3^ème^ génération (Ceftriaxone) à dose méningée (6g par jour en 2 prises en IVL). Quelques heures après l'instauration du traitement antibiotique, la patiente présente brutalement une hémiplégie droite. Un scanner cérébral de contrôle a montré des lésions hypodenses bilatérales multiples, sous corticales ne prenant pas le contraste (Figure 2). L'IRM cérébrale a révélé plusieurs lésions parenchymateuses corticales et sous-corticales temporo-fronto-pariétales gauches et temporo-pariétales droites punctiformes et linéaires en hypersignal T2, FLAIR, et diffusion d'allure ischémique (Figure 3), avec prise de contraste de la paroi des artères sylviennes de façon bilatérale en faveur d'une vascularite (Figure 4). L'angioMR veineuse ne montre pas de signes de thrombose veineuse.

**Figure 5:**
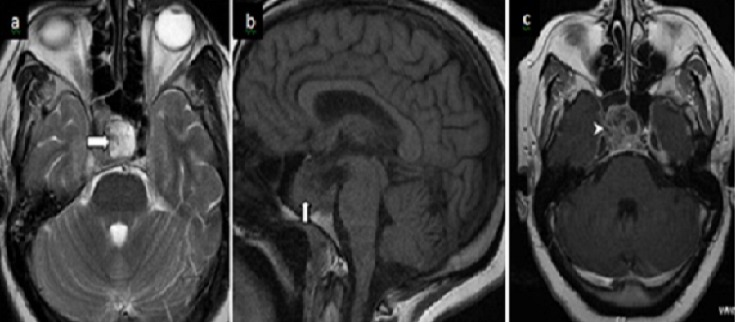
IRM cérébrale axiale T2(a), T1(b) et T1 injecté(c) en coupe axiale objectivant un processus tissulaire hypophysaire latéralisé a droite, hypointense T1, hyperintense T2, rehaussé de façon hétérogène. Noter la lyse du plancher sellaire avec extension dans le sinus sphénoidal (flèche), ainsi que l'extension caverneuse droite (tète flèche)

L’étude de la région sellaire à la TDM et à l'IRM objective un processus hypophysaire mesurant 30mm x 24mm x 22mm. Il envahit le sinus caverneux droit et englobe la carotide interne intracaverneuse qui reste perméable. Il infiltre le corps du sphénoïde et détermine une lyse du plancher sellaire avec extension dans le sinus sphénoidal. On note également une déviation la tige pituitaire à gauche (Figure 5). Un bilan hormonal a montré une hyperprolactinémie supérieure à 200 mg/ml et une insuffisance antéhypophysaire. Le diagnostic retenu est celui d'une méningo-vascularite bactérienne révélant un adénome hypophysaire à prolactine. Un traitement hormonal à base de la Cabergoline (1 mg/semaine) a été instauré avec une vaccination contre le pneumocoque comme prévention contre la méningite à pneumocoque en attendant le traitement chirurgical de la brèche ostéoméningée. La patiente s'est améliorée progressivement sous antibiothérapie et corticothérapie par voie orale (Prednisone 1mg/kg/jour) avec reprise de la conscience, récupération nette du déficit et disparition complète du syndrome méningé. Une amélioration du syndrome inflammatoire biologique (CRP à 17mg/L et Globules blancs à 9600 éléments/mm^3^). La prolactinémie est passée de 200 à 19mg/dl. Après 3 mois du traitement médical, la patiente s'est améliorée sur le plan neurologique avec persistance d'une rhinoliquorrhée. La patiente a été opérée pour fermeture de la brèche ostéodurale par voie endonasale.

## Discussion

La brèche ostéo-méningée se définit par une solution de continuité ostéoméningée entraînant une fuite du liquide céphalorachidien (LCR) de l'espace sous-arachnoïdien vers les voies naso-sinusiennes. Sa gravité principale est liée au risque d'infection du système nerveux central. La brèche ostéoméningée est le plus souvent post-traumatique (accidentelle ou iatrogène), rarement spontanée. Ainsi le diagnostic d'une rhinorrhée cérébrospinale est facile à suspecter devant l'association d'un écoulement nasal séreux à des antécédents de traumatisme crânio-facial ou de chirurgie des sinus ou de la base de crâne. Les rhinoliquorrhées secondaires sont plus fréquentes et représentent presque 96% des étiologies [3].

Parmi les étiologies des brèches ostéoméningées secondaires on retrouve la pathologie malformative (meningocéles), la pathologie inflammatoire et infectieuse (ostéomyélites de la base du crane) et la pathologie tumorale. Les processus tumoraux de la loge sellaire entraînent une augmentation de la pression intracrânienne déterminant une érosion du plancher sellaire. Les adénomes hypophysaires sont le plus souvent lentement évolutifs et pauci voire asymptomatiques. La rhinoliquorrhée est d'apparition tardive et la brèche ostéoméningée n'est révélée que secondairement après traitement chirurgical ou médical. La diminution du volume de l'adénome après traitement médical entraine une mise à nu de la brèche qui était colmatée par la tumeur avant le traitement [3]. La particularité de notre observation, c'est la survenue d'une complication infectieuse d'un adénome hypophysaire invasif par brèche de l’étage moyen de la base du crane sans que la patiente n'ait subi aucun traitement médical ou chirurgical [1, 2]. L′incidence de la méningite chez les patients porteurs d'un macroadénome hypophysaire non traité est faible et difficile à estimer puisque la plupart des données proviennent de cas rapportés ou de petites séries [2].

Lam et al. ont étudié tous les cas rapportés dans la littérature, concernant les brèches ostéoméningées associées à des adénomes hypophysaires non opérés y compris celles compliquées d'une méningite bactérienne en analysant 29 articles publiés entre 1980 et 2011. Les 52 cas publiés avaient un adénome hypophysaire avec rhinoliquorrhée. Sept cas seulement ont été compliqués par une méningite bactérienne (13%) [1, 2, 4]. Les sept cas ont été divisés en deux groupes, le premier concerne 4 cas de méningite secondaire survenant après traitement par les agonistes de la dopamine et le second concerne 3 cas de méningite primitive survenant avant tout traitement du prolactinome. Nous avons procédé à une recherche bibliographique (sur base de données Pubmed depuis 1990 jusqu'au mois de mars 2014) des cas de méningite bactérienne révélant un adénome hypophysaire survenue avant la mise en route du traitement médical ou chirurgical [1, 2, 5–14], (Tableau 1).

**Tableau 1:** Les cas de méningites bactériennes dans un contexte d'adénome hypophysaire survenant en dehors de tout traitement dans la littérature

Titre de l'article	Année	Auteur/ Référence	Nombre de cas
Occult invasive pituitary adenoma predisposing to fatal bacterial meningitis	1990	Laszewski MJ [6]	1 cas
An autopsy case of invasive pituitary adenoma (prolactinoma) with rapid fatal clinical course due to streptococcalmeningitis	1992	Onoda N [7]	1 cas
An unusual cause of acute bacterial Meningitis	2001	Stephenson [9]	1 cas
Prolactinoma with a high Adrenocorticotrop Hormone Level Caused by Meningitis	2004	S. Utsuki [8]	1cas
Meningitis as a presentation of macroprolactinoma.	2009	Honegger J [13]	1 cas
2 Cases of Meningitis as a Complication of Pituitary Adenoma	2010	Tan, H.K. [14]	1cas/2
Fulminant meningoencephalitis as the first clinical sign of an invasive pituitary macroadenoma	2010	Robert T [13]	1 cas
Suppurative meningitis: A life-threatening complication in male macroprolactinomas	2013	Farida Chentil [1]	3 cas/4
Cerebrospinal meningitis in 30-year-old man as first manifestation of pituitary macroadenoma	2013	Andrysiak [5]	1 cas
Acute aseptic meningitis as the initial presentation of a macroprolactinoma	2014	Marina Boscolo [2]	1 cas

Dans le contexte d'un adénome hypophysaire invasif, le développement de rhinorrhée de LCR en dehors de tout traitement chirurgical s'explique par la diminution de volume tumoral suite à un traitement médical de l'adénome, un infarcissement ou une hémorragie intrinsèque, et par conséquence un dévoilement de la brèche ostéodurale secondaire à l'invasion du plancher sellaire. Dans d'autres cas, l'explication peut provenir d'une invasion continue de la base, et/ou d'augmentation de la pression intracrânienne. Le scanner est pratiqué en première intention pour un tableau de rhinorrhée avec ou sans méningite. La découverte sur le scanner d'un adénome hypophysaire le plus souvent asymptomatique doit inciter à rechercher une brèche de l’étage moyen (sphénoïde) secondaire à l'adénome. Le scanner permet de mettre en évidence la lyse osseuse en l'occurrence du plancher sellaire. L'IRM offre un meilleur bilan morphologique et détermine les extensions locorégionales.

## Conclusion

L'adénome hypophysaire, même non traitée, peut potentiellement provoquer une méningite quand il est compliqué d'une brèche ostéoméningée. Ce diagnostic doit être considéré à tout moment de l'histoire d'un adénome hypophysaire et pas seulement en post-opératoire [2, 8, 10]. La prise en charge dans ces cas consiste à traiter la méningite, traiter l'adénome et fermer la brèche.
